# Metabolomic Analysis of Influenza A Virus A/WSN/1933 (H1N1) Infected A549 Cells during First Cycle of Viral Replication

**DOI:** 10.3390/v11111007

**Published:** 2019-10-31

**Authors:** Xiaodong Tian, Kun Zhang, Jie Min, Can Chen, Ying Cao, Chan Ding, Wenjun Liu, Jing Li

**Affiliations:** 1 School of Life Sciences, University of Science and Technology of China, Hefei 230026, China; tienhsiaotung@foxmail.com (X.T.); caoyingor@163.com (Y.C.); 2 CAS Key Laboratory of Pathogenic Microbiology and Immunology, Institute of Microbiology, Chinese Academy of Sciences, Beijing 100101, China; minjie00awesome@163.com (J.M.); 2008615cc@sina.com (C.C.); 3 Philips Institute for Oral Health Research, School of Dentistry, Virginia Commonwealth University, Richmond, VA 23298, USA; kzhang@vcu.edu; 4 Shanghai Veterinary Research Institute, Chinese Academy of Agricultural Sciences, Shanghai 200241, China; shoveldeen@shvri.ac.cn; 5 University of Chinese Academy of Sciences, Beijing 100049, China

**Keywords:** influenza virus, metabolomics analysis, TCA cycle, human cells, first infectious cycle

## Abstract

Influenza A virus (IAV) has developed strategies to utilize host metabolites which, after identification and isolation, can be used to discover the value of immunometabolism. During this study, to mimic the metabolic processes of influenza virus infection in human cells, we infect A549 cells with H1N1 (WSN) influenza virus and explore the metabolites with altered levels during the first cycle of influenza virus infection using ultra-high-pressure liquid chromatography–quadrupole time-of-flight mass spectrometer (UHPLC–Q-TOF MS) technology. We annotate the metabolites using MetaboAnalyst and the Kyoto Encyclopedia of Genes and Genomes pathway analyses, which reveal that IAV regulates the abundance of the metabolic products of host cells during early infection to provide the energy and metabolites required to efficiently complete its own life cycle. These metabolites are correlated with the tricarboxylic acid (TCA) cycle and mainly are involved in purine, lipid, and glutathione metabolisms. Concurrently, the metabolites interact with signal receptors in A549 cells to participate in cellular energy metabolism signaling pathways. Metabonomic analyses have revealed that, in the first cycle, the virus not only hijacks cell metabolism for its own replication, but also affects innate immunity, indicating a need for further study of the complex relationship between IAV and host cells.

## 1. Introduction

Influenza A virus (IAV), a member of the Orthomyxoviridae family, is a negative-sense, single-stranded, enveloped, segmented RNA virus [1,2]. IAV usually infects epithelial cells of the upper and lower respiratory tracts, including the nasal mucosa, trachea, and lungs, with no evident symptoms during the early phase of infection [3,4]. Once an influenza virus invasion occurs, innate immunity is activated, and interferons are secreted by host cells to limit the early viral proliferation [3]. Then, adaptive immunity is activated by other cytokines produced during viral infection. However, in some cases, highly pathogenic influenza viruses induce cytokine storms, a consequence of excessive production of cytokines and interferon, resulting in infections and even death [5].

To facilitate virus replication in the host cells, IAV has evolved strategies to block the innate and adaptive immune responses of the host cells and seize organelles from host cells to synthesize a large number of metabolites required for viral reproduction, as well as energy for the packaging of the virus [6,7]. Enveloped, non-enveloped, DNA and RNA viruses share lipid metabolites in their replication cycles to induce the formation of new cytoplasmic membrane structures, which contribute to the replication and packaging of the viral genome [8,9,10]. Lipid metabolism also can block the innate immune response of host cells to ensure the large-scale replication of the virus. Therefore, IAV infection is linked closely to metabolism, and the proliferation of the virus also is inseparable from the host metabolism. This changing trend in small molecule metabolites may serve as a characterization of host–pathogen interactions to monitor immune status.

Although significant progress has been made toward an anti-influenza virus drug discovery, including M2 ion channel blockers, neuraminidase inhibitors, and polymerase inhibitors [11], challenges posed by drug toxicity and viruses with genetic resistance remain a serious problem [12,13,14,15]. Previous research demonstrated the metabolic effects of influenza virus infection in Madin–Darby canine kidney (MDCK) cells, displaying the intra- and extra-cellular metabolite profiling upon IAV infection [16,17]. Little is known, however, about the systemic metabolic dynamics during the early stage of virus infection. During our study, we analyze changes in metabolism upon influenza virus infection in human cells during the first infectious cycle via metabolomics. Early metabolite analysis will throw new light on the activation of the innate immune metabolism. We believe that the results of this work will elucidate the activation of innate immunity to viral infection from the perspective of the host and provide new control strategies for the development of novel drugs and the treatment and prevention of influenza virus infection.

## 2. Materials and Methods

### 2.1. Cell Culture and Viral Preparation

Human lung carcinoma epithelial cells (A549), MDCK cells and mouse lung epithelium (MLE-12) cells were grown in Dulbecco’s modified Eagle’s medium (DMEM; Gibco) supplemented with 10% fetal bovine serum (FBS, Gibco) in 5% CO_2_ at 37 °C. The IAV A/WSN/33 (H1N1) was propagated at 37 °C for 72 h in allantoic cavity-specific pathogen-free embryonated eggs at 10 days of age. Virus titers were determined by a plaque assay. Virus stocks were stored at −80 °C until use.

### 2.2. Plaque Assay

MDCK cells were seeded in 12-well plates, infected with serial dilutions of the virus in serum-free DMEM supplemented with 4 μg/mL of l-1-tosylamido-2-phenyethyl chloromethyl ketone (TPCK)-treated trypsin for 2 h, and then washed with phosphate-buffered saline (PBS). The cells were covered with Modified Eagle’s Medium containing 1% agarose (AMRESCO) and 2 μg/mL of TPCK-treated trypsin. The plates were allowed to solidify at 4 °C for 5 min and incubated upside-down at 37 °C. Following 72 h, viral titers were determined by counting the visible plaques.

### 2.3. Virus Infection In Vitro and In Vivo

When the A549 cells reached high confluence (>95%), they then were cultured for 4 h in serum-free DMEM, compared with controls under identical culture conditions, and infected with WSN at a multiplicity of infection (MOI) of 0.1, 1, and 5. The virus inoculums were removed by washing with PBS and incubation in DMEM for the indicated times in 5% CO_2_ at 37 °C. The infected cells were collected at 0 h, 8 h, and 16 h and stored at −80 °C.

The A/WSN/33 (H1N1) virus titer was determined by plaque assays. Groups of six 6–8-week-old female BALB/c mice were intranasally inoculated with 50 μL of 5000 p.f.u of virus diluted in phosphate-buffered saline (PBS). Mock-infected control animals were inoculated intranasally with 50 μL PBS. Animals that showed signs of severe disease and weight loss >30% of their initial body weight were considered moribund and were sacrificed humanely according to animal ethics guidelines. Five mice from each group were euthanized at 0 h, 12 h, 24 h, and 48 h and necropsies were performed. The lung tissue samples were homogenized in PBS with antibiotics in a homogenizer and used to determine the viral titers using the plaque assay. The lung tissue and serum were divided into three portions, used for an enzyme-linked immunosorbent assay (ELISA) and a metabolite concentration test, respectively.

### 2.4. Immunofluorescence Assay

Cells were cultured overnight in 24-well plates. Prior to the assays, cells were cultured for 4 h in a serum-free medium and then infected with WSN at a MOI of 0.1. Cells (500 μL) were collected at 0 h, 2 h, 5 h, and 8 h, washed with PBST, fixed in 4% paraformaldehyde, and stored at 4 °C overnight. Samples then were blocked with 4% bovine serum albumin (BSA) and stained with anti-influenza A virus nucleoprotein (NP) antibody (1:500). The secondary antibody (1:200) was fluorescein isothiocyanate (FITC) -conjugated goat anti-rabbit IgG, followed by 4′,6-diamidino-2-phenylindole (DAPI) staining for 15 min. Samples then were observed using a model Leica SP8 confocal laser scanning ﬂuorescence microscope (Olympus). A549 cells and MDCK cells also were collected at 0 h, 12 h, 16 h, and 24 h, and infected with WSN at a 0.1, 1, and 5 MOI (Figure S1).

### 2.5. Sample Preparation, ELISA, and Metabolomics Analysis

The cells were washed twice with pre-cooled PBS and then lysed with 1 mL of methanol/acetonitrile/water (2:2:1, *v*/*v*) by vortexing twice for 30 min at 4 °C. The lysates then were incubated for 1 h at −20 °C, followed by centrifugation at 13,000× *g*/min for 15 min at 4 °C. The supernatants were collected and stored at –80 °C for further analysis. Metabolic concentration was determined by an ELISA assay according to the manufacturer’s instruction.

### 2.6. Data Acquisition through LC-MS Analysis

Samples were separated on an Agilent 1290 Infinity ultra-high-pressure liquid chromatography–quadrupole time-of-flight mass spectrometer (UHPLC-Q-TOF MS), with a column temperature of 25 °C, flow rate of 0.3 mL/min, and injection volume of 2 μL.

The mobile phase contained A (water, 25 mM ammonium acetate, and 25 mM ammonia) and B (acetonitrile). The gradient elution procedure was as follows: 0 min–1 min, 95% B; 1 min–14 min, decreased linearly from 95–65%; 14 min–16 min, B was decreased linearly from 65–40%; 16 min–18 min, B was maintained at 40%; 18 min–18.1 min, B was increased linearly from 40–95%; 18.1 min–23 min, B was maintained at 95%. Samples were placed in a 4 °C autosampler throughout the process. To avoid the effects of instrument detection signal fluctuations, continuous analysis of samples was performed in random order. QC samples were inserted into the sample queue to monitor and evaluate the stability of the system and the reliability of the experimental data.

The electrospray ionization (ESI) positive and negative ion modes were used for mass spectrometer (MS) detection. The samples were separated by ultra-high-pressure liquid chromatography (UHPLC) and subjected to MS using a Triple TOF 5600 mass spectrometer (ABSCIEX). The ESI source conditions were as follows: Ion Source Gas1 (Gas1): 60, Ion Source Gas2 (Gas2): 60, Curtain gas (CUR): 30, source temperature: 600 °C, IonSapary Voltage Floating (ISVF) range −5500 V to 5500 V; TOF MS scan m/z range: 60 Da–1000 Da, production scan m/z range: 25 Da–1000 Da, TOF MS scan accumulation time 0.2 s/spectra, production scan accumulation time 0.05 s/spectra; Information Dependent Acquisition (IDA) was obtained and adopted high sensitivity mode, Declustering potential (DP) range −60 V to 60 V; Collision Energy range 20 eV to 50 eV; IDA was set to exclude isotopes with 4 Da, candidate ions to monitor per cycle 6.

### 2.7. Statistical Analysis

XCMS software (https://xcmsonline.scripps.edu/index.php) was used to analyze the raw data for peak alignment, calibration, and retention time peak area extraction. Metabolite structure identification used a method of accurate mass matching (<25 ppm). Ion peaks with missing values >50% in the data group were deleted. SIMCA-P 14.1 (Umetrics, Umea, Sweden) was used to establish a statistical model [18]. The data were preprocessed by Pareto-scaling for multidimensional statistical analysis, including unsupervised principal component analysis (PCA) [19,20], supervised partial least squares discriminant analysis (PLS-DA) [21], and orthogonal partial least squares discriminant analysis (OPLS-DA) [22]. Single-dimensional statistical analysis included Student’s *t*-test and variation multiple analyses, and the PCA maps, volcano maps, and cluster maps were generated with the R program.

### 2.8. Differential Metabolite Analysis and Functional Pathway Analysis

Via the Variable Importance for the Projection (VIP), the characteristics of metabolite expression patterns were used to mine the differential metabolites with biological significance. During our study, VIP >1 was selected as the screening standard, and the differences between the groups initially were screened. Univariate statistical analysis was used to confirm significant differences in metabolites. Differential metabolites were identified by adjustments of the *p*-value for multiple testing at both VIP >1 and univariate statistical analysis *p* < 0.05.

To identify the altered metabolic pathways involved during influenza virus infection, the differential metabolites were subjected to the statistical tool MetaboAnalyst 4.0 (www.metaboanalyst.ca), which is a web-based service that provides online visual statistical analysis [23]. Data were uploaded to KEGG (www.kegg.jp) and HMDB 4.0 (www.hmdb.ca) for more information to identify significantly altered pathways [24,25,26,27]. All these programs support a variety of complex statistical calculations and high-quality graphic rendering capabilities that require copious computing resources.

## 3. Results

### 3.1. Rapid Replication of IAV in the Early Stages of Infection in Human Cells

**Figure 1:**
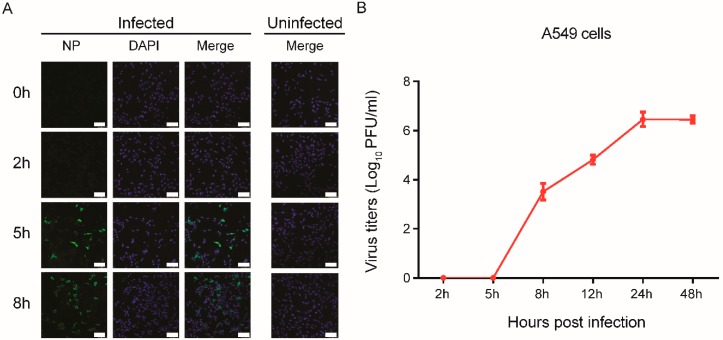
A549 cells were infected with A/WSN/1933 at different time points. (**A**) Immunofluorescence staining of A549 cells post-infection with A/WSN/1933. Infected cells were distributed in four wells of a 24-well plate at a MOI of 0.1. The influenza virus NP protein was analyzed with FITC-conjugated antibody (left), and the nuclei were examined using DAPI staining (middle). Uninfected control is shown on the right. Scale bar, 100 μm. (**B**) Growth curve of IAVs in A549 cells. The cells were infected with A/WSN/1933 virus (MOI of 0.1). The supernatants were collected at the indicated time points, and viral titers were determined by plaque-forming units.

To confirm virus replication in A549 cells, the cells were infected with A/WSN virus at a MOI of 0.1, and virus replication was analyzed. The ratio of infected cells also was identified by measuring viral intracellular NP using immunofluorescence microscopy analysis. We found that the number of infected cells at 8 h was greater than that of cells infected at 2 h and 5 h (Figure 1A), and that virus titers in the A549 cell progressively increased until reaching a peak at 24 h post-infection, indicating more efficient virus replication within 24 h post-infection (Figure 1B). Consistent results were observed in A549 cells infected with A/WSN/1933 and analyzed at different time points, and the virus production was comparable in a single-cycle infection, while infected cells at a MOI of 1 or 5 displayed a higher cell death (Figure S1).

### 3.2. Characteristic Metabolites in Response to Virus Infection

**Figure 2:**
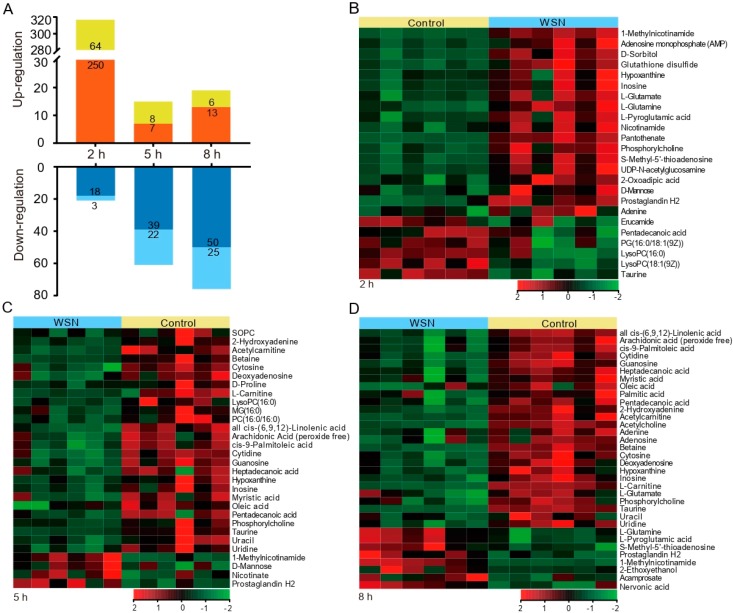
Identification and characterization of altered metabolites after IAV infection. (**A**), Bar graph showing a large number of metabolite changes. The *X*-axis represents the time point, and the negative log_10_ of the *p*-value on the *y*-axis. The metabolites with log_2_ fold changes >1 or <−1 and −log_10_ *p* > 1.3 were significantly different. Red (positive ion modes) and yellow (negative ion modes) indicate up-regulated, while dark blue (positive ion modes) and light blue (negative ion modes) indicate down-regulated. (**B**–**D**), A549 cells were infected with A/WSN/1933 viruses at a MOI of 0.1 for 2 h (**B**), 5 h (**C**), and 8 h (**D**). Total metabolites were extracted and used for metabolomic analysis. The expression values shown in shades of green and red indicate gene levels below and above the median expression value across all the samples (log_2_, from −2 to +2), respectively. Each row is a differential metabolite, and each column represents a replicate of a group.

Metabolite isolates were prepared individually from both WSN virus- and mock-infected A549 cells. To identify the functions of the characteristic metabolites during viral infection, univariate analysis was performed to analyze the total metabolite profiles in uninfected or WSN-infected A549 cells. Volcano plots in Figure S2 show all differentially expressed metabolites were identified. The variations in metabolites were correlated with different time points, and changes in up-regulated metabolites were more abundant at 2 h post-infection, while the down-regulation of metabolites was more significant at 8 h post-infection (Figure 2A).

To compare metabolite expression profiles at 2 h, 5 h, and 8 h post-infection, we filtered metabolites with fold analysis, calculating the 50 differentially expressed metabolites (Table 1). Shown in the heat map diagrams in Figure 2B–D, we depict the upregulated and downregulated metabolites in A549 cells responding to WSN virus infection induced at different time points, indicating the various metabolic influences induced by virus infection.

### 3.3. KEGG Pathway Enrichment Analyses Based on Metabolites

**Figure 3:**
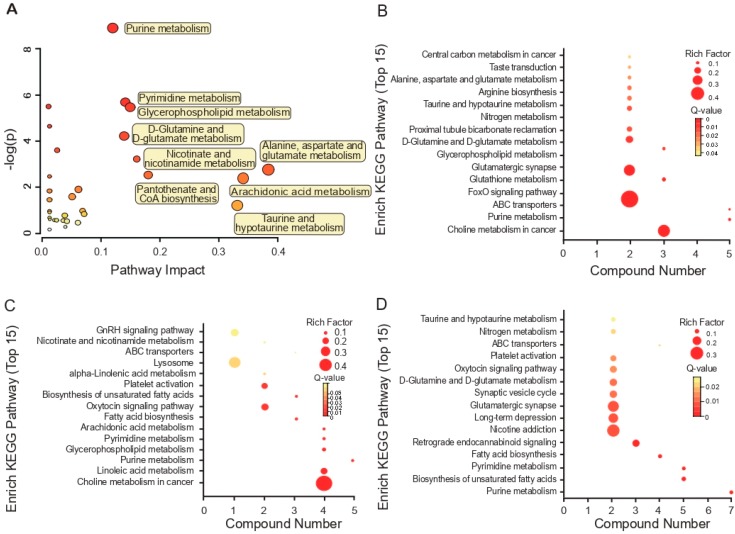
Top 15 enriched pathways based on characteristic metabolites in A/WSN/1933-infected cells. Pathway analysis allowed the construction of a scatter plot of KEGG pathway enrichment statistics for characteristic metabolites following A/WSN/1933 infection of A549 cells. (**A**) Global metabolic disorders of the most relevant pathways induced by A/WSN/1933 were revealed using MetaboAnalyst 4.0. “Pathway Impact” means that the selected metabolites conducted topological analysis of the metabolic pathway according to their different positions in the metabolic pathway. The corresponding score is shown on the *X*-axis, and the *p*-value (*Y*-axis) of the metabolic pathway enrichment analysis is selected as the most valuable metabolic pathway, (**B**–**D**). Rich factor is the ratio of the number of differentially expressed genes noted in the pathway terms to all metabolite numbers found in this pathway term. We selected the top 15 of the KEGG enrichment results as a reference. “Compound number” is the compounds here referring to the ones in Table 1. A greater Rich Factor indicates higher intensity. To control the false discovery rate (FDR), we used *q* = 0.05 to correct the *p*-value of the metabolites, ranging from 0 to 1. A lower *q*-value indicates higher intensity.

To identify the biological interactions of metabolites and determine important functional networks upon WSN infection in human cells, we analyzed the statistically enriched metabolites, listing the top 20-fold changes by the absolute value of the log_2_ scale obtained from the A549 cell lines (Figure 3A); we mapped the metabolites with altered expression into their KEGG pathways. We present the results of the top 30 pathways activated by the WSN virus in A549 cells in Table 2. Upon WSN virus infection, the most significantly activated cellular metabolite process was purine metabolization.

We also mapped metabolites identified at individual time points into the KEGG pathway database to further explain the individual function analysis. The top 15 enriched pathways in WSN-infected A549 cells are summarized in Figure 3. Two hours post-infection, ABC transporters and the FoxO signaling pathway, which are in the biological process category, were regulated most significantly by WSN infection (*Q* < 0.05 and *p* <0.01) (Figure 3B). During the next 6 h, choline metabolization in cancer and taurine, and hypotaurine metabolization were associated highly with the responses to WSN infection in A549 cells (Figure 3B,C).

### 3.4. Metabolite Correlation Network Diagram Analysis

**Figure 4:**
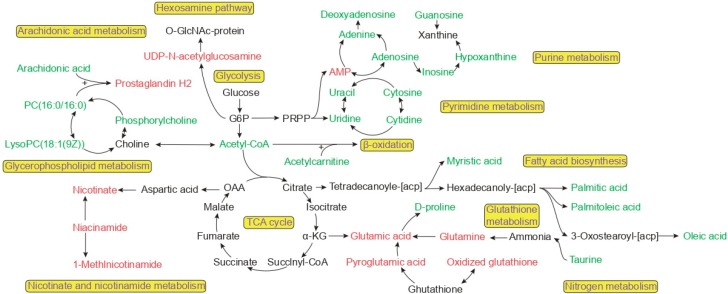
Schematic of metabolic pathways influenced by IAV infection. The pathways depicted here are indicative of numerous cellular metabolic pathways. Glycerophospholipid metabolism, glutathione metabolism, fatty acid biosynthesis, the hexosamine pathway, glycolysis, and purine metabolism pathways are highlighted. The metabolites with red (upregulated) and green (downregulated) labels are significantly altered metabolites in A/WSN/1933.

We also used MetaboAnalyst 4.0 analyses to reveal the possible functions of the identified unique metabolites in cell samples. Examining the differential unique metabolites in the WSN group relative to glutathione metabolization and purine metabolization in A549 cells, we found the most enriched biological processes to be related mainly to the TCA cycle, arachidonic acid metabolization, and the hexosamine pathway (Figure 4), with purine metabolization and fatty acid biosynthesis as the most significant molecular functions.

### 3.5. Trends in Key Metabolites by Box Plots of Different Times by Infection

By assembling box plots of selected metabolites across our experimental time, the differential expression profiles of the metabolites were validated. Although minor differences were observed in the different times due to their intrinsic differences, the results of these analyses demonstrated the key relative regulation of metabolites (Figures S3–S5).

**Figure 5:**
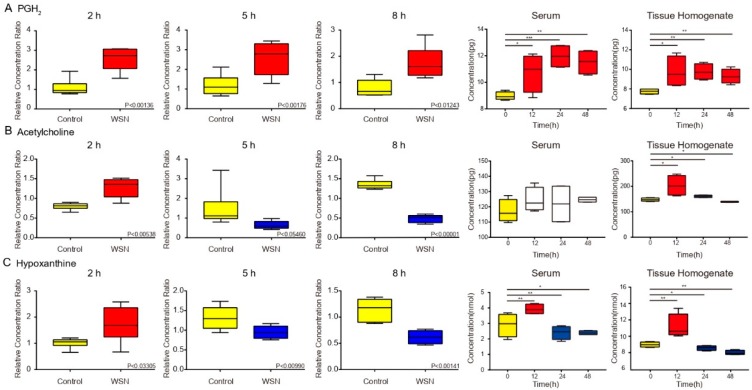
Key metabolites by box plots of different times by infection. Metabolite concentration changes in (**A**) PGH2, (**B**) acetylcholine, (**C**) hypoxanthine, extracted from human cells, serum, and lung tissue post-infection were determined by using each peak intensity ratio. Each *p*-value is filled on the box plot with the metabolite name, and the maximum/minimum value and dispersion of the data are illustrated in GC/MS chromatograms.

The common regulative metabolites (PGH, O-acetylcholine, and hypoxanthine) induced by WSN infections are linked to elevated morbidity and mortality in humans [28]. Figure 5A shows the expression of the most regulative metabolites differed in infected A549 cells between 2 h post-infection and the next 6 h. However, the levels of PGH and O-acetylcholine, as well as hypoxanthine, were decreased markedly in WSN-infected A549 cells at 5 h post-infection. These findings suggest a remarkable initiation of the response of the host metabolic levels and capacity in WSN-infected A549 cells. To further confirm these results in vivo mice were intranasally inoculated with 50 μL of 5000 p.f.u of virus or PBS as a negative control. The lung tissue homogenates and serum samples were detected using an ELISA assay. Consistent results were observed in PGH2 and hypoxanthine expression. According to the metabolomic analysis data, acetylcholine displayed similar expression in the lung tissue, while the expression in serum was not changed significantly (Figure 5A). Additionally, we observed similar results in mouse lung epithelium (MLE-12) cells (Figure S6). The viral lung titers of the mice infected with WSN also are displayed in Figure S7.

## 4. Discussion

Influenza virus infection is linked inextricably to metabolism, and the proliferation of the virus is inseparable from the host metabolism. The mouse-adapted WSN virus, which is recognized as a neurovirulent strain, also can cause severe lung hypoxemia and pulmonary edema in mice [29]. Previous research demonstrated that blunting the cytokine storm significantly alleviated the syndrome of animals infected with WSN virus [5,30]. Though the prospect of blunting over-abundant innate immune response is enticing, little is known about the activation of an innate immune metabolism during early virus infection and the potential metabolites modulating immune response and virus replication. During our study, we identified >50 differential metabolites by exploring the metabolism and metabolic characteristics of early viral infection, established through the integration of statistical analyses and metabolic networks. Host metabolic changes upon influenza virus infection play a key role in regulating virus replication.

Influenza viruses can utilize the host’s energy metabolism for their own replication. Our study found no significant changes in intermediate metabolites in the TCA cycle during the first replication cycle (prior to 8 h). We conclude that the enzyme in the TCA cycle is still active. We observed the same phenomenon in the TCA cycle in the PR8-infected MDCK cell model over the first 10 h [16]. The IAV usually leads to apoptosis, which is caused by damage to the mitochondrial membrane after infection [31]. Apoptosis leads to more severe metabolic disorders, destroying cellular respiration. Apoptosis-related gene transcription levels were downregulated within 8 h prior to WSN infection [32]. Therefore, we believe that mitochondria remain intact in morphology and function during the first replication cycle of an influenza virus, and no apoptosis occurs. Concurrently, an increase in glutamate content was observed in glutathione metabolization, a strategy in which cells maintain high levels of oxidized coenzymes under high pressure to maintain an energy metabolism balance. During the first replication cycle, the mitochondrial energy supply is maintained by up-regulating the glutamate content to maintain TCA cycle stability to complete viral replication. 

Theoretically, even if all a metabolite were disappeared in infected cells, expression of the metabolite would become 80–90% in this MOI 0.1 condition. However, our data shows that many metabolites were down-regulated several times, while our speculation was related to the regulation of uninfected cells by infected cells. Previous studies have found that virus infection between cells is a spatial process, depending on where the virus is at the infection time point. Infected cells gradually activate the antiviral immunity of surrounding uninfected cells through cytokines such as interferon [33,34,35]. Our study concentrated on the first cycle of virus replication, there was no progeny virus production. Therefore, we speculated that the infected cells, centered on the infected cells, communicated with the surrounding signals and produced the same metabolic changes, instructing the uninfected cells to enter the antiviral state, thus leading to such changes in metabolites.

We also found the negative regulatory effect of influenza virus on the metabolic pathway for fatty acids. According to our data, the relative abundance of myristic acid, palmitic acid, palmitoleic acid, and oleic acid was decreased. Fatty acids have been known to play a dual role in an influenza virus metabolism [36,37,38,39,40]. Previous research demonstrated the virulence of an influenza virus is enhanced by palmitoylation of the cysteine residues in the M2 protein in vivo, although this palmitoylation process is not necessary in the formation of IAV in vitro [41]. Additionally, other research suggests that palmitoyl-oleoyl-phosphatidylglycerol (POPG) [42], a minor component of pulmonary surfactants, effectively regulates the innate immune system. The presence of POPG significantly attenuates influenza virus-induced IL-8 production and apoptosis in human bronchial epithelial cells. During early infection, this POPG thus serves to activate the innate immune system to inhibit influenza virus replication.

Host nucleotides and their derivatives, consumed upon influenza virus replication, are important small molecules in cells involved in signal transduction [43,44,45,46], energy cycling [9,47] and the synthesis of genetic material [48]. Li et al., demonstrated that UDP-N-acetylglucosamine was used as a substrate for the hexosamine biosynthetic pathway (HBP) to glycosylate MAVs, an important signaling adaptor protein in the innate immune signaling pathway [49]. Thus, HBP plays an important role in the antiviral effect of innate immunity by targeting MAVs protein. Our results confirmed that the relative amount of UDP-*N*-acetylglucosamine increased during the first replication cycle of the virus, which may increase the glycosylation of MAVS, helping it to form prion-like aggregates to activate an antiviral response in innate immunity after viral infection. We also observed that purine metabolization changed significantly during the first cycle. Purine plays an important role in the biological processes of cells [50,51], Chandler et al., reported lung tissue was taken for metabolomics analysis at 10 days by IAV infection to obtain a decrease in AMP content; our data further enhances previous research that AMP content increased within 2 h by the first cycle of viral replication [52]. This may indicate that AMP plays an irreplaceable role as a core component in the metabolism of purine [53]. A sharp increase in AMP may increase the ratio of ATP/AMP, thereby activating the AMPK pathway [54,55,56,57], followed by beta-oxidation of fatty acids and glycolysis, providing more energy to the cell. The basic carbon skeleton is required for the synthesis of viruses. Here we demonstrate that influenza viruses widely use nucleotides and derivatives thereof as synthetic substrates during replication and, therefore, nucleotide starvation effectively can modulate immune responses, thereby reducing the efficiency of viral replication [58].

Furthermore, prostaglandins are reported to be used by IAV to achieve immune escape. Prostaglandin H2 was upregulated in our study. PGH2 is the first intermediate in the biosynthesis of all prostaglandins, which can be converted into PGE2 and PGD2 with biological activity [59,60]. PGE2 is expressed in macrophages during influenza infection, and inhibiting PGE2 can promote the aggregation of macrophages into the lungs and produce interferon [59]. However, to expand the infection of the influenza virus, DC cell migration is inhibited by PGD2 [60]. Thus, the blocking of prostaglandin synthesis during early infection leads to the accelerated activation of immune cells in the lungs to suppress infection.

Taken together, the metabolic activity of the virus in the early stage of infection plays a critical role in evading the host’s innate immunity and preparing a large number of substrates for its replication and proliferation. Therefore, virus infection can be targeted in the early stages of virus reproduction based on its characteristics. Accompanying the analysis of metabolomic studies, broad-spectrum antiviral drugs against post-infection lipid metabolization have been developed [8,13]. This affirms that metabolomics can serve as a mature research method to regulate influenza virus infection and contribute to the prevention and treatment of influenza [55].

## 5. Conclusions

To identify host cell responses to influenza-infected host cells, we used metabolomic analysis to identify differentially expressed metabolites between uninfected controls and IAV-infected A549 cells. We found that, compared to the control group, the IAV-infected group displayed a large amount of altered metabolic activity, with significant differences found in 50 discrete metabolites. These were distributed mainly in purine metabolization, lipid metabolization, and glutathione metabolization, which accelerate the replication speed of the virus for the first replication cycle of the influenza virus, but also causes innate immunity to monitor metabolic changes. To summarize, our research suggests novel approaches for the future development of immune metabolism studies and provides evidence for further confirmation of the complex regulatory mechanisms between IAV and host cells.

## Figures and Tables

**Table 1:** Summary of differentially expressed metabolites data.

No.	Time (h)	Metabolites	Formula	M/Z	Mr	HMDB	PubChem	KEGG
1	2	1-Methylnicotinamide	C_7_H_9_N_2_O	137.07	137.1	HMDB0000699	457	C02918
2	2	Pantothenate	C_9_H_17_NO_5_	218.1035	219.2	HMDB0000210	988	C00864
3	2	Sorbitol	C_6_H_14_O_6_	181.0722	182.1	HMDB0000247	5780	C00794
4	2	L-Glutamine	C_5_H_10_N_2_O_3_	147.0754	146.1	HMDB0000641	5961	C00064
5	2	S-Methyl-5’-thioadenosine	C_11_H_15_N_5_O_3_S	298.0959	297.3	HMDB0001173	439176	C00170
6		Oxidized glutathione	C_20_H_32_N_6_O_12_S_2_	613.1575	612.6	HMDB0003337	975	C00127
7	2	LysoPC (18:1(9Z))	C_26_H_52_NO_7_P	522.3537	521.7	HMDB0002815	16081932	C04230
8	2	Taurine	C_2_H_7_NO_3_S	126.0208	125.1	HMDB0000251	1123	C00245
9	2	Phosphorylcholine	C_5_H_15_NO_4_P	184.0724	184.2	HMDB0001565	1014	C00588
10	2	Uridine diphosphate-N-acetylglucosamine	C_17_H_27_N_3_O_17_P_2_	608.087	607.4	HMDB0000290	445675	C00043
11	2	LysoPC (16:0)	C_24_H_50_NO_7_P	496.3379	495.6	HMDB0010382	460602	C04230
12	2	L-Glutamic acid	C_5_H_9_NO_4_	148.0596	147.1	HMDB0000148	33032	C00025
13	2	Pyroglutamic acid	C_5_H_7_NO_3_	130.0488	129.1	HMDB0000267	7405	C01879
14	2	Niacinamide (Niacinamide)	C_6_H_6_N_2_O	123.0541	122.1	HMDB0001406	936	C00153
15	2	Adenosine monophosphate (AMP)	C_10_H_14_N_5_O_7_P	348.0695	347.2	HMDB0000045	6083	C00020
16	2	Inosine	C_10_H_12_N_4_O_5_	269.0871	268.2	HMDB0000195	6021	C00294
17	2	Hypoxanthine	C_5_H_4_N_4_O	137.0446	136.1	HMDB0000157	790	C00262
18	2	Adenine	C_5_H_5_N_5_	136.0609	135.1	HMDB0000034	190	C00147
19	2	Erucamide	C_18_H_19_NO_4_	338.3408	313.3	HMDB0029365	5280537	C02717
20	2	Prostaglandin H2	C_20_H_32_O_5_	351.2177	352.5	HMDB0001381	445049	C00427
21	2	Oxoadipic acid	C_6_H_8_O_5_	141.0171	160.1	HMDB0000225	71	C00322
22	2	D-Mannose	C_6_H_12_O_6_	179.0562	180.1	HMDB0000169	18950	C00159
23	2	PG (16:0/18:1(9Z))	C_40_H_77_O_10_P	747.5194	749.0	HMDB0010574	52941750	/
24	2	Pentadecanoic acid	C_15_H_30_O_2_	241.2175	242.4	HMDB0000826	13849	C16537
25	5	Uridine	C_9_H_12_N_2_O_6_	245.0758	244.2	HMDB0000296	6029	C00299
26	5	L-Carnitine	C_7_H_15_NO_3_	162.1115	161.2	HMDB0000062	2724480	C00318
27	5	Deoxyadenosine	C_10_H_13_N_5_O_3_	252.1082	251.2	HMDB0000101	13730	C00559
28	5	PC (16:0/16:0)	C_40_H_80_NO_8_P	778.536	734.0	HMDB0000564	452110	C00157
29	5	2-Hydroxyadenine	C_5_H_5_N_5_O	152.0557	151.1	HMDB0000403	76900	/
30	5	Uracil	C_4_H_4_N_2_O_2_	111.0198	112.1	HMDB0000300	1174	C00106
31	5	MG (0:0/16:0/0:0)	C_19_H_38_O_4_	331.2837	330.5	HMDB0011533	123409	/
32	5	Adenosine	C_10_H_13_N_5_O_4_	268.1033	267.2	HMDB0000050	60961	C00212
33	5	D-Proline	C_5_H_9_NO_2_	116.0694	115.1	HMDB0003411	8988	C00763
34	5	Nicotinate (Nicotinic acid)	C_6_H_5_NO_2_	124.0383	123.1	HMDB0001488	938	C00253
35	5	PC (18:0/18:1(9Z)) (SOPC)	C_44_H_86_NO_8_P	832.582	788.1	HMDB0008038	24778825	C00157
36	5	L-Acetylcarnitine	C_9_H_17_NO_4_	204.1221	203.2	HMDB0000201	1	C02571
37	5	Cytidine	C_9_H_13_N_3_O_5_	244.0919	243.2	HMDB0000089	6175	C00475
38	5	Cytosine	C_4_H_5_N_3_O	112.0494	111.1	HMDB0000630	597	C00380
39	5	Guanosine	C_10_H_13_N_5_O_5_	284.098	283.2	HMDB0000133	6802	C00387
40	5	Betaine	C_5_H_11_NO_2_	118.0852	117.1	HMDB0000043	247	C00719
41	8	Acetylcholine	C_7_H_16_NO_2_	146.1164	146.2	HMDB0000895	187	C01996
42	8	2-Ethoxyethanol	C_4_H_10_O_2_	151.0955	90.1	HMDB0031213	8076	C14687
43	8	Palmitoleic acid	C_16_H_30_O_2_	253.2176	254.4	HMDB0003229	445638	C08362
44	8	Oleic acid	C_18_H_34_O_2_	281.2488	282.5	HMDB0000207	445639	C00712
45	8	Arachidonic acid	C_20_H_32_O_2_	303.2332	304.5	HMDB0001043	444899	C00219
46	8	Myristic acid	C_14_H_28_O_2_	227.2022	228.4	HMDB0000806	11005	C06424
47	8	Heptadecanoic acid	C_17_H_34_O_2_	269.2486	270.5	HMDB0002259	10465	/
48	8	Nervonic acid	C_24_H_46_O_2_	365.3424	366.6	HMDB0002368	5281120	C08323
49	8	Palmitic acid	C_16_H_32_O_2_	255.2333	256.4	HMDB0000220	985	C00249
50	8	Acamprosate	C_5_H_11_NO_4_S	180.0335	181.2	HMDB0014797	71158	/

**Table 2:** Top pathways activated by H1N1-WSN virus in A549.

No.	Name of Pathway	Total	Expected	Hits	−log_10_ *p*-Value
1	Purine metabolism	92	1.6053	8	8.9037
2	Pyrimidine metabolism	60	1.0469	5	5.6818
3	Nitrogen metabolism	39	0.68052	4	5.4648
4	Glycerophospholipid metabolism	39	0.68052	4	5.4648
5	Fatty acid biosynthesis	49	0.85501	4	4.6447
6	D-Glutamine and D-glutamate metabolism	11	0.19194	2	4.2132
7	Glutathione metabolism	38	0.66307	3	3.6014
8	Nicotinate and nicotinamide metabolism	44	0.76776	3	3.2223
9	Alanine, aspartate and glutamate metabolism	24	0.41878	2	2.7426
10	Pantothenate and CoA biosynthesis	27	0.47113	2	2.5348
11	beta-Alanine metabolism	28	0.48857	2	2.4715
12	Arachidonic acid metabolism	62	1.0818	3	2.387
13	Arginine and proline metabolism	77	1.3436	3	1.9036
14	Galactose metabolism	41	0.71541	2	1.8366
15	Fructose and mannose metabolism	48	0.83756	2	1.5918
16	Taurine and hypotaurine metabolism	20	0.34898	1	1.2115
17	Aminoacyl-tRNA biosynthesis	75	1.3087	2	0.97063
18	Fatty acid elongation in mitochondria	27	0.47113	1	0.96782
19	alpha-Linolenic acid metabolism	29	0.50602	1	0.91228
20	Lysine biosynthesis	32	0.55837	1	0.83755
21	Amino sugar and nucleotide sugar metabolism	88	1.5355	2	0.77921
22	Butanoate metabolism	40	0.69796	1	0.67663
23	Histidine metabolism	44	0.76776	1	0.61188
24	Primary bile acid biosynthesis	47	0.82011	1	0.56861
25	Lysine degradation	47	0.82011	1	0.56861
26	Glycine, serine and threonine metabolism	48	0.83756	1	0.55507
27	Fatty acid metabolism	50	0.87246	1	0.52922
28	Cysteine and methionine metabolism	56	0.97715	1	0.46024
29	Tryptophan metabolism	79	1.3785	1	0.27865
30	Porphyrin and chlorophyll metabolism	104	1.8147	1	0.16712

## Supplementary Materials

The following are available online at https://www.mdpi.com/1999-4915/11/11/1007/s1, Figure S1. Immunofluorescence staining of A549 cells post-infection with A/WSN/1933 at MOI of 0.1, 1, or 5. Figure S2. Volcano plots of metabolic changes of virus infection at different time points. Figure S3. Box plots of the 2 h metabolic changes of virus infection in A549 cells. Figure S4. Box plots of the 5 h metabolic changes of virus infection in A549 cells. Figure S5. Box plots of the 8 h metabolic changes of virus infection in A549 cells. Figure S6. The concentration of metabolites in mouse lung epithelial cells (MLE-12). (A) Metabolic changes in PGH2, (B) changes in acetylcholine metabolism, and (C) changes in hypoxanthine metabolism. Figure S7. The viral lung titers of the mice infected with WSN.
