# Supplementary figures and images for: Metabolomic Analysis of Influenza A Virus A/WSN/1933 (H1N1) Infected A549 Cells during First Cycle of Viral Replication
Source: Viruses. 2019 Oct 31;11(11):1007. doi: 10.3390/v11111007 (PMC6893833; doi:10.3390/v11111007)

0.1 MOI

1 MOI

5 MOI

0 h

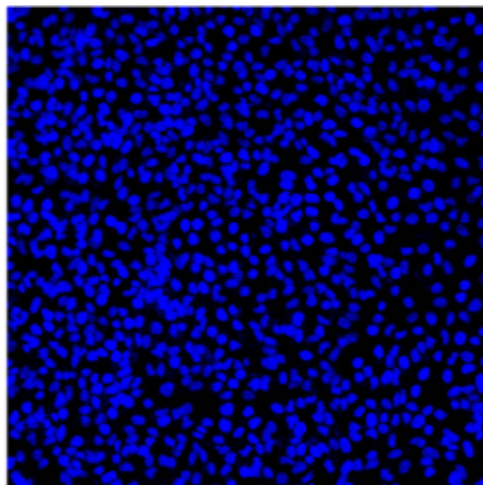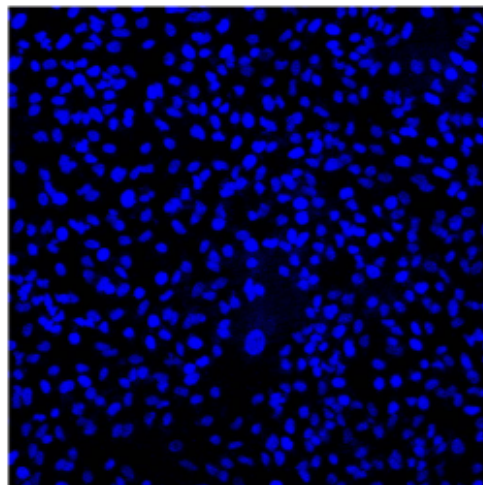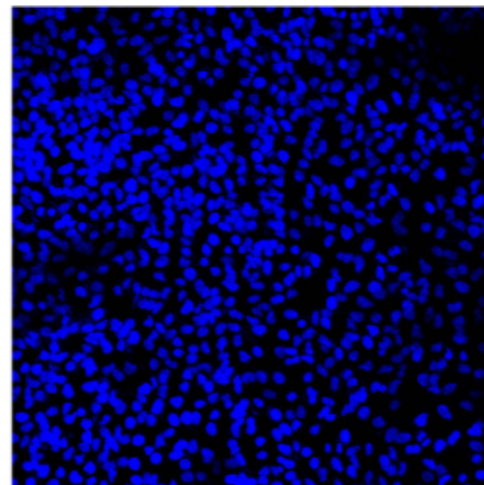

8 h

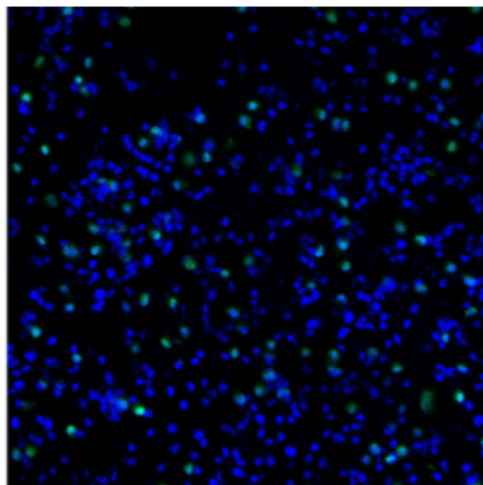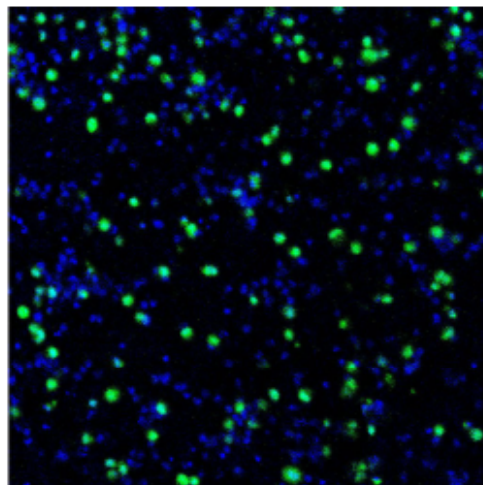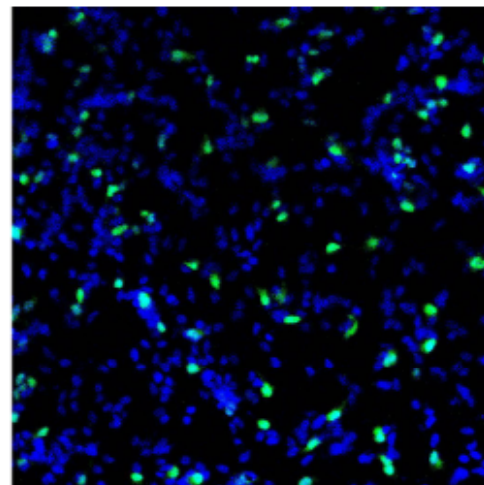

16 h

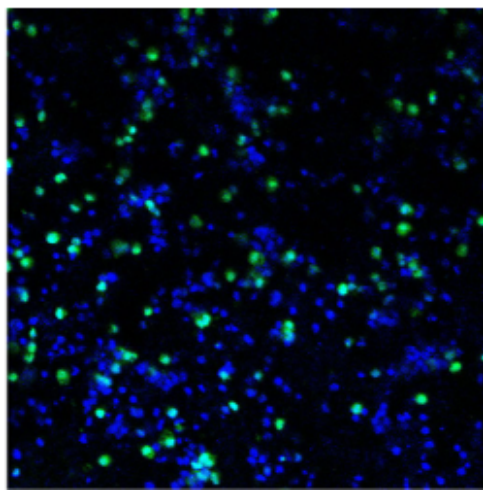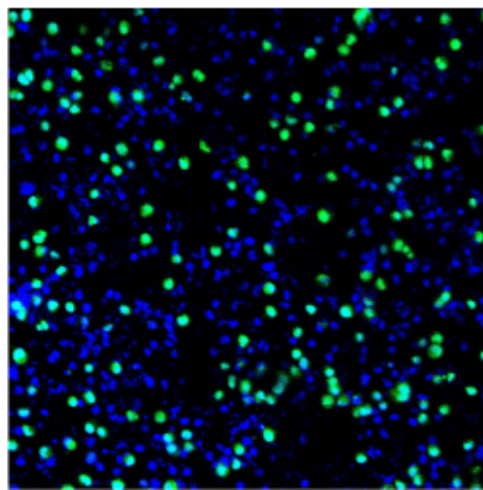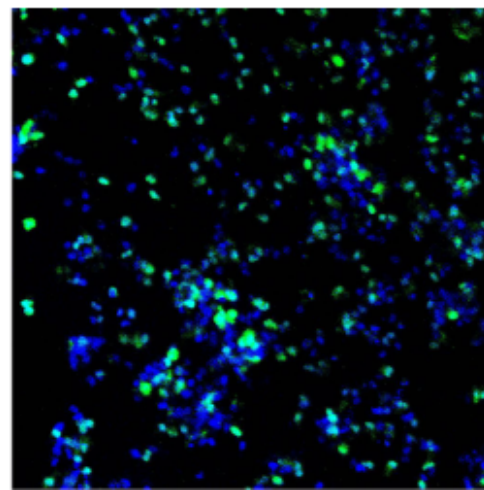

A

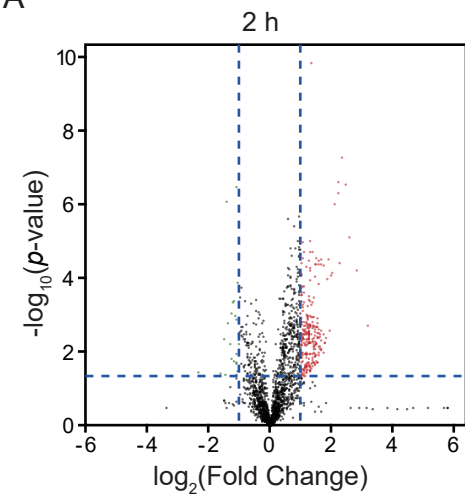

B

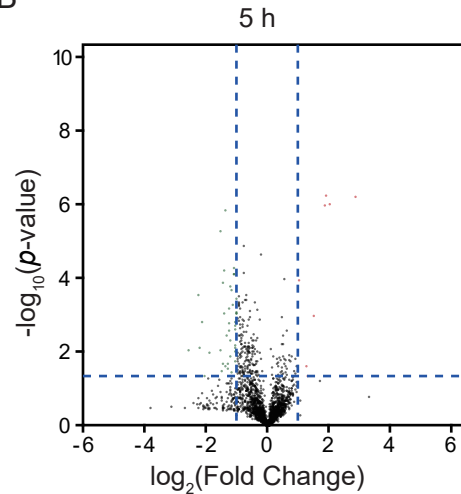

C

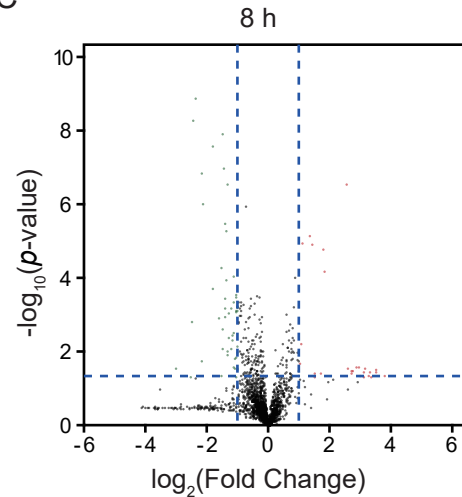

D

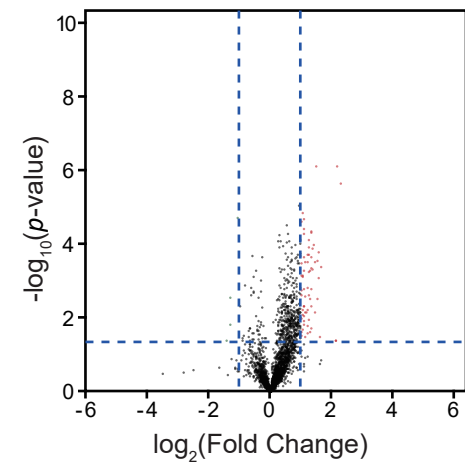

E

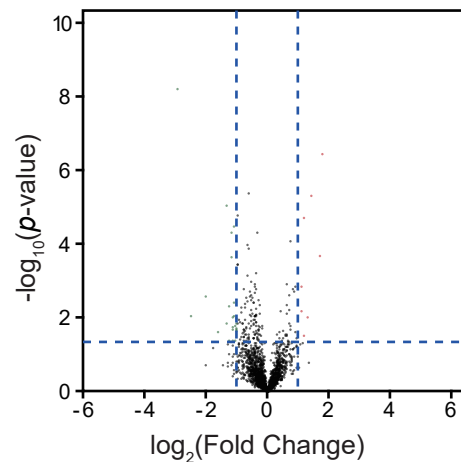

F

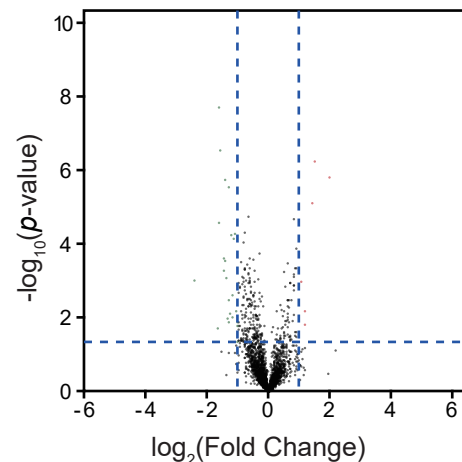

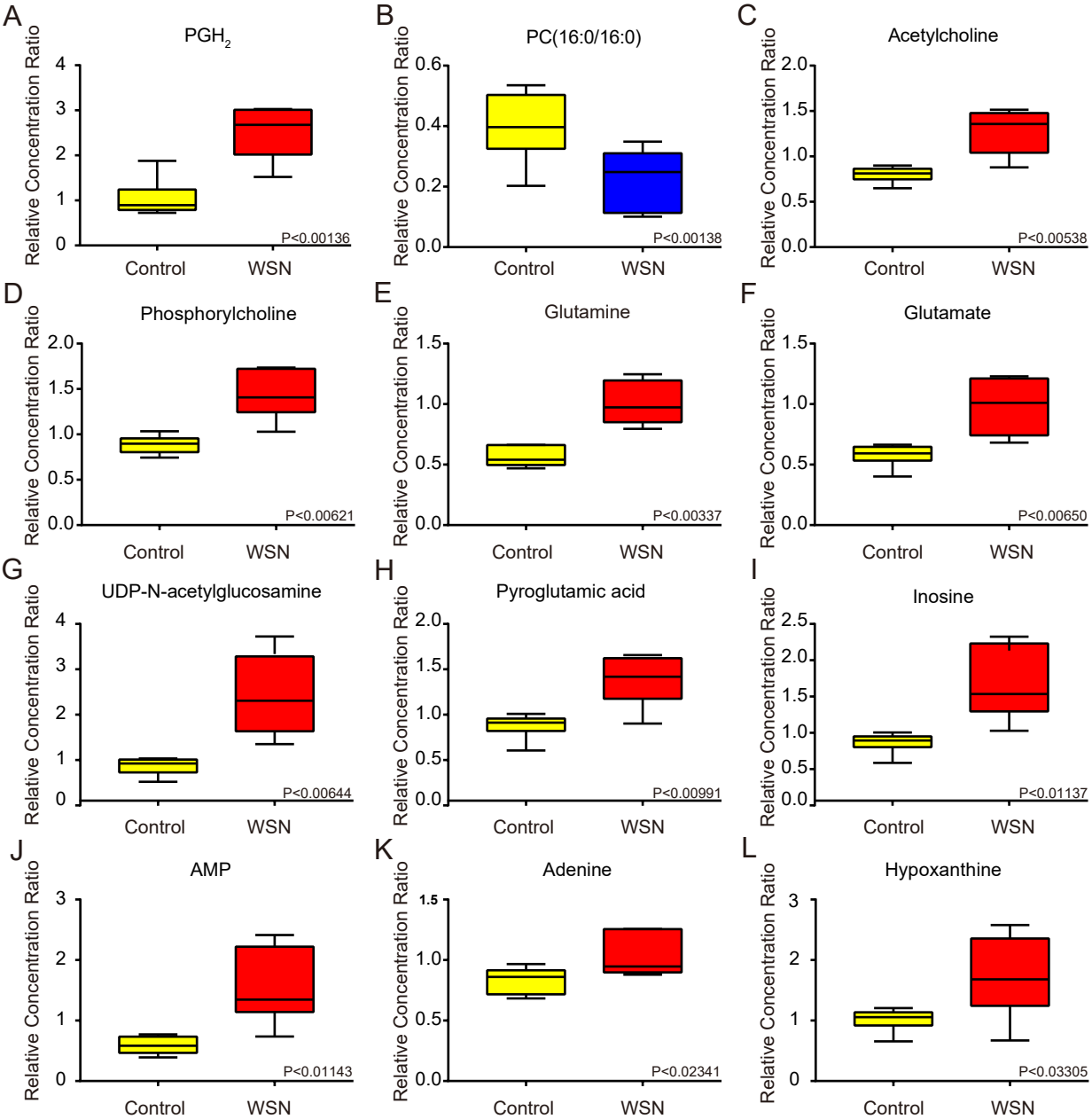

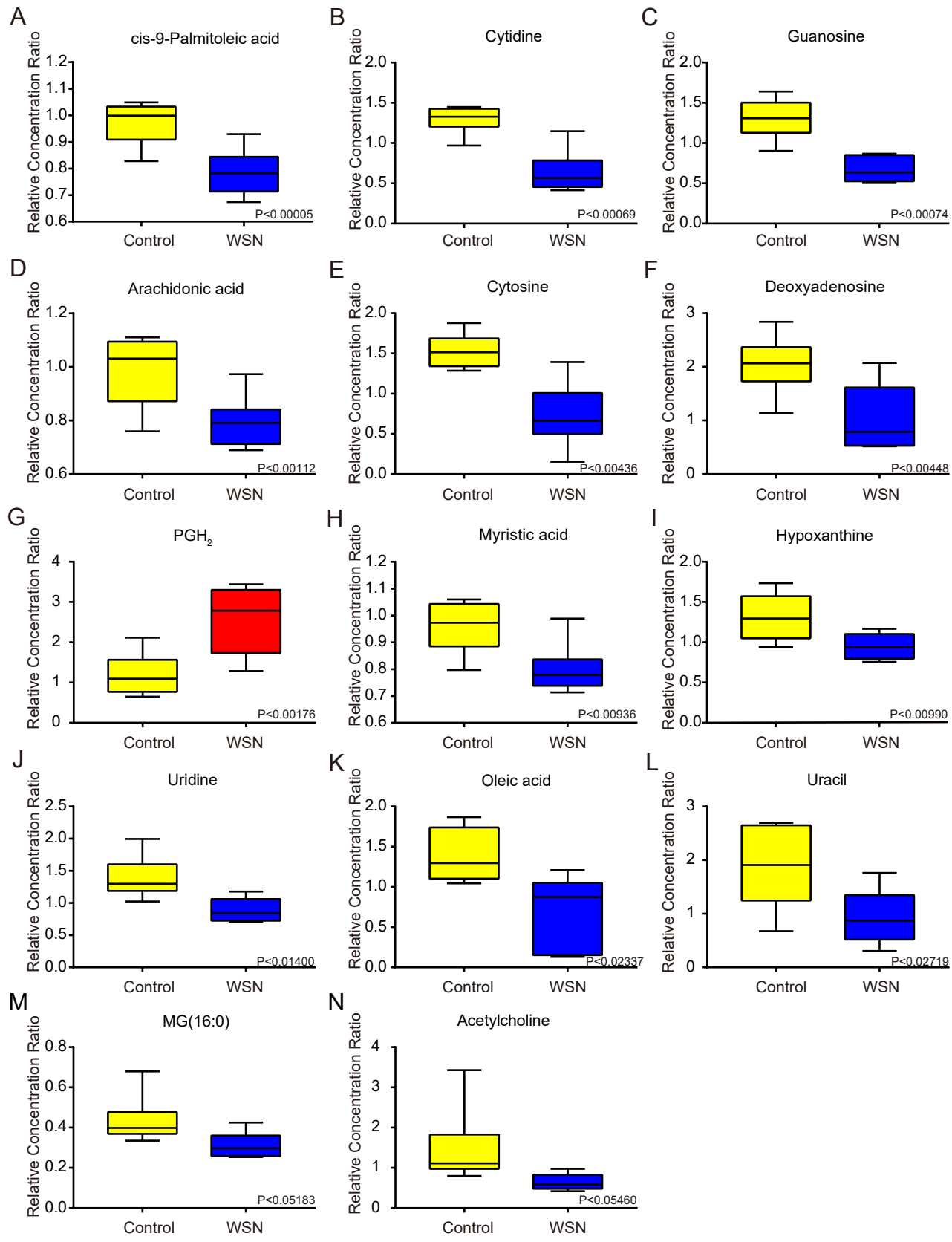

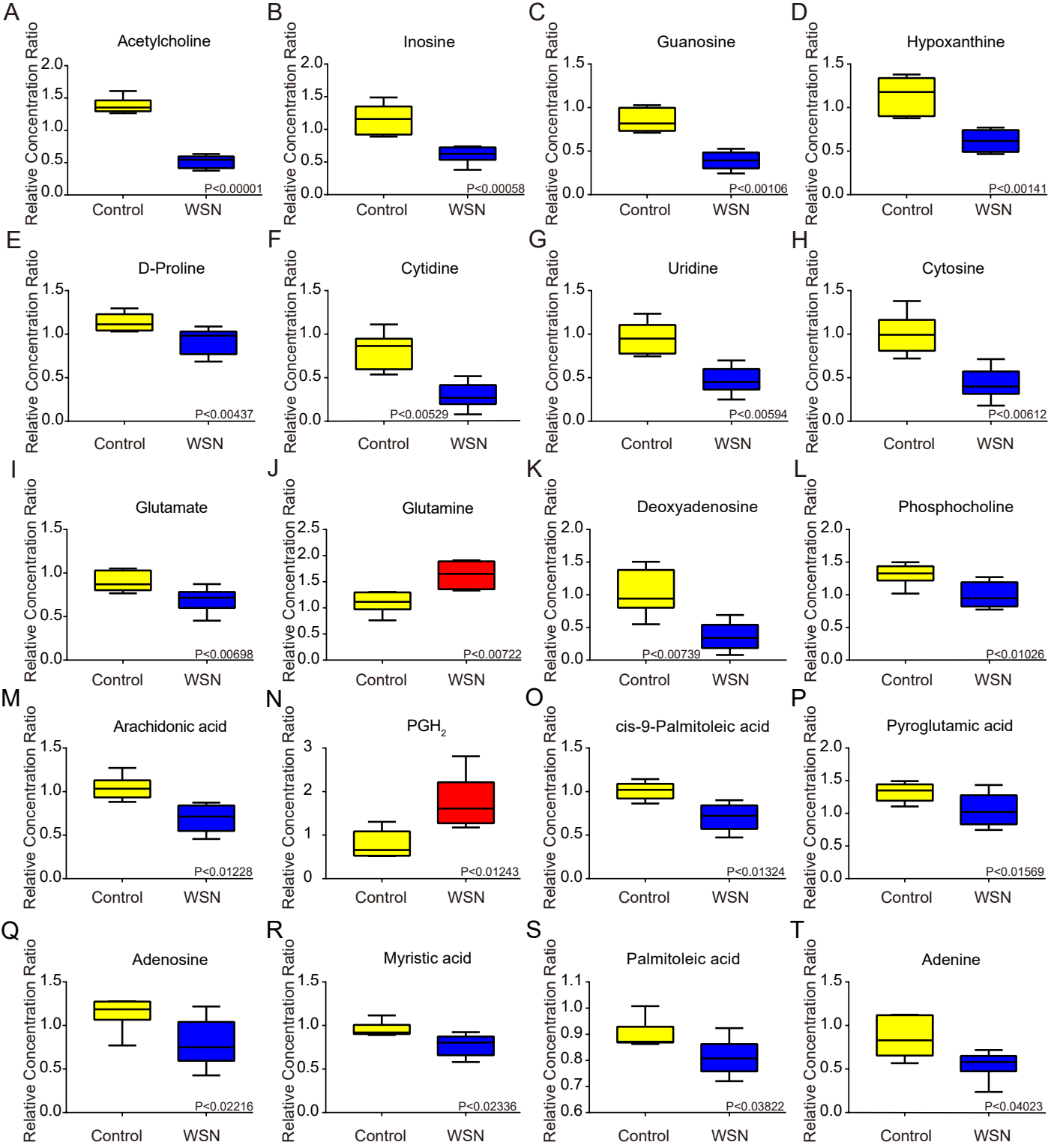

A

PGH2

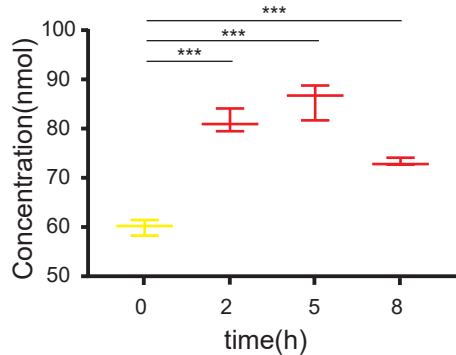

B

Acetylcholine

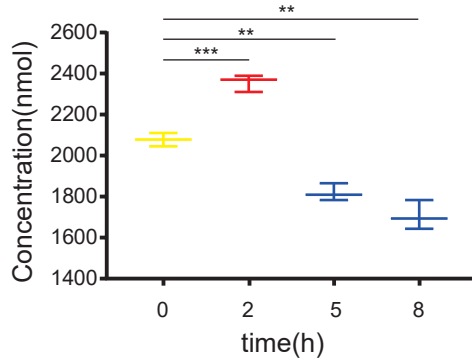

C

Hypoxanthine

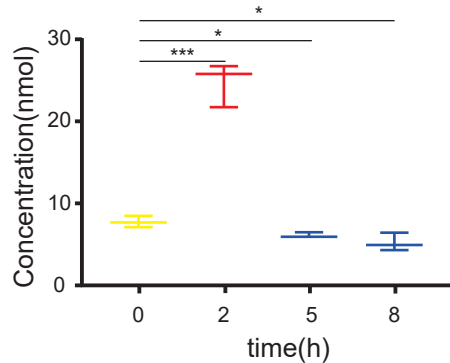

Supplement: Supplementary file 1 [file viruses-11-01007-s001.pdf]
